# Finding new immune regulatory genes by ENU mutagenesis

**DOI:** 10.1186/1479-5876-10-S3-I6

**Published:** 2012-11-28

**Authors:** Anselm Enders, Hannes Bergmann, Mehmet Yabas, T Dan Andrews, Belinda Whittle, Mathew Field, Bhavani Balakishnan, Yovina Sontani, Lisa Miosge, Geoff Sjollema, Stuart Read, Ed Bertram, Chris C Goodnow

**Affiliations:** 1 Rammaciotti Immunisation Genomics Laboratory, John Curtin School of Medical Research, Australian National University, Canberra, Australia; 2 Immunogenomics Laboratory, John Curtin School of Medical Research, Australian National University, Canberra, Australia; 3 Australian Phenomics Facility, Australian National University, Canberra, Australia

## Introduction

The Thousand Genomes Project has revealed the extraordinary scale of human genetic variation such as each person inherits ~12,000 protein-changing single nucleotide variations (SNVs) including up to 100 premature STOP codons creating an immense challenge to investigate the physiological consequence of this type of genetic variation in experimental animals.

## Aim

To develop a new strategy to meet this need by using whole exome capture, massively parallel DNA sequencing and computational analysis to sensitively and specifically identify 40-50 *de novo* mis-sense SNV mutations spread across the genome of individual offspring from ENU-mutagenized C57BL/6 mice [1].

## Results

In this presentation we demonstrate how direct sequencing of animals with independent defects in B cell development resulted in the identification of the causative mutation both in genes of previously known and unknown function without meiotic mapping. This approach has resulted in the identification of a new phospholipid transport pathway that is crucial for normal B cell development in the bone marrow [2] and the realization that an endopeptidase of previously unknown *in vivo* function is essential for normal processing of MHC invariant chain and terminal B cell and DC maturation.

By sequencing animals from defined strains and also founder animals of ENU mutant pedigrees two generations before the phenotypic screens we have started to build a database of missense mutations containing currently close to 7000 mutations in mouse pedigrees actively breeding and immediately available for experimental analysis. All data is made available from the missense variant database (pb.apf.edu.au/phenbank).

## Conclusion

This approach transforms mammalian experimental genetics and opens up an unparalleled source for experimental models of human disease and validation of human disease associated SNVs.
